# The Role of Financial Fragility and Financial Control for Well-Being

**DOI:** 10.1007/s11205-021-02627-5

**Published:** 2021-02-15

**Authors:** Piotr Bialowolski, Dorota Weziak-Bialowolska, Eileen McNeely

**Affiliations:** Sustainability and Health Initiative (SHINE), Department of Environmental Health, Harvard T.H. Chan School of Public Health, 665 Huntington Avenue, Suite G28, Boston, MA 02115 USA

**Keywords:** Emotional well-being, Physical well-being, Social well-being, Financial fragility, Financial control

## Abstract

Financial fragility is recognized as a substantial issue for individual well-being. Various estimates show that between 46 and 59% of American adults are financially fragile and thus vulnerable in terms of their well-being. We argue that the role of financial control in shaping well-being outcomes—despite being less recognized in the literature than the role of financial fragility—is equally or even more important. Our study is a longitudinal cohort study that made use of observational data. Two waves of the Well-Being Survey data from 1448 U.S. adults were used in the analysis. Impacts of financial fragility and financial control on 17 well-being outcomes were examined, including emotional well-being (nine outcomes), physical well-being (four outcomes), social well-being (two outcomes), in addition to an unhealthy days summary measure and the flourishing index. Financial fragility was shown to be on average less influential for the well-being outcomes than financial control. Our results suggest that financial control plays a protective role for complete well-being. Less evidence in support of a harmful role of financial fragility for well-being is provided. Tests for moderation effects revealed no interaction between financial control and financial fragility within our sample, indicating that financial control did not modify the relationship between financial fragility and well-being.

## Introduction

The aggregated U.S. household debt amounts to $13.5 trillion (Federal Reserve Bank of New York 2019), i.e., 80% of the U.S. GDP (International Monetary Fund 2019). It is currently at an all-time high, putting American debtholders in a very precarious position and increasing their vulnerability to external shocks (e.g., COVID-19 pandemic). These high levels of debt were previously hypothesized to be linked with a lack of financial planning skills, poor financial management, and detrimental consumption behaviors resulting from beliefs that material things can lead to happiness (Donnelly et al. 2012; O’Neill et al. 2017).

Despite having on average one of the highest disposable personal incomes in the world, American adults often remain financially fragile. Various estimates show that between 46% (Gupta et al. 2018) and 59% (*Report on the Economic Well-Being of U.S. Households in 2017*, 2018) of American adults would be incapable of paying for a $400 emergency expense given their available financial resources or lack thereof. Even an unexpected expense of $100 could not be met by 16% of American adults and 34% would be unable to come up with $2000 within the following month (Gupta et al. 2018). These numbers raise questions about financial literacy (Allgood and Walstad 2016; Ward and Lynch 2019), money management (i.e., budgeting, saving, and investing Donnelly et al. 2012; O’Neill 2015]), and healthy spending of U.S. adults. They also call into question the role of one’s financial situation for complete well-being.

We argue that more evidence is needed to evaluate the impact of an individual’s financial situation on complete well-being. Findings from the literature are often narrowly focused on emotional well-being or mental health only. This hinders addressing the link between one’s financial situation and other domains of well-being beyond the emotional one. Moreover, most available evidence is based on cross-sectional data. This, in turn, leads to issues with reverse causality^1^ that render results inconclusive or overestimated (Weziak-Bialowolska et al. 2020).

Evidence for the financial control and well-being link is ambiguous. On the one hand, it has been shown that people feeling in control of their non-financial spheres are less likely to fall into excessive debt (Tokunaga 1993; Wahlund and Gunnarsson 1996) and save more (Cobb-Clark et al. 2016). On the other hand, they are also more likely to take financial risks (Kesavayuth et al. 2018), which might increase variance of their financial outcomes and lead to lower well-being outcomes in a substantial group of unlucky or unskilled investors. Hence, more empirical evidence on the role of financial control is needed.

This article aims to provide a broader perspective and examine how financial fragility and financial control influence emotional, physical, and social well-being. It also responds to the call of Strömbäck et al. (2017) to look more closely into which cognitive and non-cognitive skills [such as personality traits or “patterns of thought, feelings, and behavior” (Borghans et al. 2008)] influence well-being. Striving to evaluate earlier findings, within our sample we assess the extent to which financial fragility has a detrimental effect on well-being [research question 1 (RQ1)], and what the scale (if any) of positive effect of financial control on well-being can be identified (RQ2). We also examine within our sample whether financial control reduces the detrimental impact of financial fragility on well-being (RQ3). In other words, we expect that individuals with higher levels of financial control would be less affected by financial fragility simply because, first, they will be less likely to become financially fragile, and second, detrimental effects of financial fragility would be moderated by their conviction that, through their own actions, they are more in control of future outcomes.

This study offers three novel contributions. First, it is the first to investigate the impact of financial fragility on physical, emotional, and social well-being among a sample of U.S. residents, also considering a potential moderating effect of financial control. Second, by analyzing the role of financial control, it focuses on the positive aspect of financial management rather than merely on mitigating the damage of financial distress and adverse financial circumstances for well-being and health. In this sense, by studying resilience in the financial domain and the role of factors associated with resilient financial behaviors, our study responds to the call of Seligman (2008) to promote positive health instead of merely preventing ill-health. Third, our use of longitudinal data, which by virtue of the depiction of the logical temporal sequence of cause and effect, permits more evidence for causal interpretation of results.

## Theoretical Background

Excessive debt and financial hardship can lead to a variety of negative outcomes such as housing instability (Burgard et al. 2012; Cannuscio et al. 2012), poverty and consequential social exclusion (D’Alessio and Iezzi 2013), decreased economic well-being (D’Alessio and Iezzi 2013; Wałęga and Wałęga 2021), and alcohol, tobacco and drug addictions (Angel 2016; Richardson et al. 2013; Turunen and Hiilamo 2014). Research also supports the claims of detrimental impact of financial hardship on emotional health (Bridges and Disney 2010; Selenko and Batinic 2011; Sweet et al. 2013), specifically on psychological stress (Gathergood 2012), psychological well-being (Białowolski et al. 2019; Brown et al. 2005; Tay et al. 2017), mental disorders (Emami 2010), depression and suicidal thoughts (Białowolski et al. 2019; Fitch et al. 2007; Richardson et al. 2013; Turunen and Hiilamo 2014). A negative impact of financial hardship was also noted for physical health (Nettleton and Burrows 1998; Sweet et al. 2013), self-reported health (Blázquez and Budría 2015; Kyriopoulos et al. 2016; Sweet et al. 2013; Turunen and Hiilamo 2014), obesity (Emami 2010; Münster et al. 2009), and blood pressure (Sweet et al. 2013).

There are numerous approaches to reflect and measure the state of household financial affairs. They can either focus on negative components such as financial distress, financial stress, financial hardship, financial vulnerability or financial fragility (Anderloni et al. 2012; Brunetti et al. 2012; Lusardi et al. 2011; Worthington 2006) or examine these phenomena from a positive perspective by assessing financial capability, financial well-being and financial wellness (Białowolski and Węziak-Białowolska 2014; Brüggen et al. 2017; Gerrans et al. 2014; Joo 2008; Prawitz et al. 2006). Despite these dualistic perspectives, most of the indicators for financial conditions are negative and refer to a financial burden. Specifically, they refer to either self-reported and perceived debt burden, perceived financial hardship, opinions on repayment difficulties or financial stability, evaluation of the level of difficulty in making ends meet and other qualitative assessments of financial problems (Anderloni et al. 2012; Białowolski and Węziak-Białowolska 2014; Cobb-Clark and Ribar 2009; Keese 2012; McCarthy 2011), or objective monetary assessments such as an amount of resources at a bank or a postal account (Brunetti et al. 2012), debt service ratio and debt-to-income ratio (Brown and Taylor 2008; Dey et al. 2008) or relation of financial burdens to housing costs (Georgarakos et al. 2010; May and Tudela 2005).

Looking at financial behavior from the perspective of secure flourishing in life (VanderWeele 2017b; Weziak-Bialowolska et al. 2019a), particular attention should be devoted to financial indicators responsible for long-term well-being and flourishing. The concepts of financial fragility and financial capability seem to serve this purpose. Financial fragility reflects a lack of resources to cope with a potential, unexpected expense (Brunetti et al. 2012; Lusardi et al. 2011; Worthington 2003). Although it is argued to capture sensitivity of a household to fall into arrears and insolvencies in reaction to the amount of lending and to macroeconomic shocks (Jappelli et al. 2008), it does not contain components of foresight and thus does not inform about the ability to sustain a financial position in the long run. Therefore, a more comprehensive measure of financial capability has been also proposed (Atkinson et al. 2006; Noctor et al. 1992). It refers to making informed judgements and effective decisions in the realm of money management (Noctor et al. 1992) and being capable of managing one’s finances, coping with income reduction, and having financial resources to meet unexpected expenditures (Brunetti et al. 2012; Lusardi et al. 2011). Thus not only is financial capability strongly linked to financial literacy (Hoelzl and Kepteyn 2011), but it also includes elements of financial control. Specifically, among five domains of financial capability identified by Atkinson et al. (2006), four relate entirely to financial control (i.e., oversight over financial issues, planning for the future, choosing financial products wisely, and seeking information), while only one partially relates to financial distress and fragility (i.e., being able to make ends meet).

The financial control aspect of financial capability has not gained sufficient scientific attention yet, despite being a likely factor for promoting well-being. Financial control can be conceptualized as financial locus of control, which is defined as ‘the perception of personal control over financial affairs’ (Furnham 1986, p. 37). The financial locus of control adds an additional dimension to the traditional concept of locus of control that, in turn, represents someone’s generalized attitudes, beliefs, or expectations regarding the nature of the causal relationship between her behavior and its consequences (Buddelmeyer and Powdthavee 2016; Rotter 1966). Theoretical considerations distinguish internal (i.e., a belief that what happens in one’s life stems from one’s own actions) and external (i.e., a belief that what happens in one’s life is beyond one’s control since it results from external factors, e.g., fate, chance, other people [Buddelmeyer and Powdthavee 2016]) locus of control. Empirical studies have shown that people with high internal locus of control are more determined in their pursuit of goals, react to problems in a more constructive manner, actively search for solutions, and have relatively good coping skills (Butterfield 1964; Headey 2008). Instead, people with high external locus of control are more likely to bow to pressure and often rely on emotional support (Buddelmeyer and Powdthavee 2016; Butterfield 1964). This might suggest that locus of control in the financial domain has a strong linkage with well-being.

Although the evidence of the role of financial control for well-being is limited, importance of the locus of control for financial behaviors has already been established. Individual differences in self-control have been shown to influence financial and economic behavior and financial well-being (Lunt and Livingstone 1991; Strömbäck et al. 2017). Those who feel in control, that is—reveal a high perceived internal locus of control, were found to manage their finances more prudently, to be less likely to acquire excessive debt (Tokunaga 1993; Wahlund and Gunnarsson 1996) and to save more (Cobb-Clark et al. 2016). Financial control seems also especially important in light of the mounting evidence of various behavioral factors leading households to over-indebtedness or under-savings (Białowolski 2019; Karlan and Zinman 2012). Specifically, the need for financial control is particularly apparent for persons affected by hyperbolic discounting (also referred to as a myopia), that is demonstrating a tendency of time-inconsistent economic choices with high preference for present consumption.^2^ Not only has hyperbolic discounting been shown to be prevalent among regular consumers (Angeletos et al. 2001) but also to be related to the lack of self-control (Laibson 1997).

## Material and Methods

### Data

In this study two waves of the SHINE^3^ Well-Being Survey were used. The survey was part of a project aimed at examining the effects of a broad impact financial incentive on well-being in a community that was identified by the 2015 Community Well-Being Rankings and Access to Care (Gallup Healthways Well-Being Index 2015) as experiencing considerable financial hardship, and also physical well-being deficiencies.^4^

Invitation letter was sent in June 2018 to all members of a credit union residing in one of the North Carolina counties in the U.S. 4083 persons responded to the survey. In September 2019 they were invited to participate in the second wave of the survey. There were 1448 respondents who provided responses in both waves and were subject to the analysis. Participation was voluntary. Informed written consent was obtained from all participants. Protocols for recruitment and participation were reviewed and approved by the Institutional Review Board of the Harvard T.H. Chan School of Public Health. Descriptive statistics of the sample and of the county population are presented in Table 1.

**Table 1:** Baseline descriptive statistics

Characteristic	Sample	County^‡^
*Gender*		
Male	35.0%	47.3%
Female	65.0%	52.7%
*Age*^†^		
18–24	3.7%	12.2%
25–34	18.9%	17.1%
35–44	19.9%	16.2%
45–54	20.4%	17.0%
55–64	21.0%	16.6%
> 64	16.2%	20.9%
*Race*		
White	76.7%	66.6%
African American	16.6%	27.5%
Alaskan Native	1.3%	0.9%
Asian	2.3%	2.6%
Other	3.1%	2.4%
Education (bachelor’s degree or higher)	64.2%	33.0%
Married	57.2%	–
Having children under 18 years old who currently live with the respondent	61.9%	–
Taking care of an elder(s)	5.1%	–
*Labor market status*		–
Employed	78.8%	–
Looking for a job	2.3%	–
Not working	18.9%	–
Community engagement in the last 12 months (yes)	39.6%	–
Volunteering (hours spent in the last 30 days)—mean (SD)	8.2 (14.6)	–
Voting in the last presidential election in 2016 (yes)	90.5%	–
Religious service attendance (at least once a week)	36.1%	–
Sport activity (moderate to vigorous–intensity sports, fitness or recreational activities in the last 5 days [min])—mean (SD)	106.6 (133.6)	–
Number of alcoholic drinks consumed in a typical week		–
0	40.0%	–
1–5	42.2%	–
More than 5	17.8%	–
Smoking (yes)	7.5%	–
Worrying about being able to meet monthly living expenses (1 = do not ever worry; 10 = worry all the time)—mean (SD)	3.3 (3.0)	–
Worrying about food, safety and housing (1 = do not ever worry; 10 = worry all the time)—mean (SD)	2.4 (2.7)	–

^†^Since detailed county-level data on age structure were not available, we used North Carolina state-level data as a proxy. The comparison of the 10-year age groups between county and the state indicated that these structures are very similar

https://censusreporter.org/profiles/05000US37067-forsyth-county-nc/, visited July 3rd, 2020; Table B01001

^‡^U.S. Census Bureau (2018). *American Community Survey 1-year estimates.* Retrieved from *Census Reporter Profile page for Forsyth County, NC* http://censusreporter.org/profiles/05000US37067-forsyth-county-nc/, visited July 3rd, 2020

The sample selection process was not different than in most of the state-of-the-art surveys with the exception that instead of selecting a random sample, we approached the whole population of customers of the federal credit union (Bialowolski et al. 2021). However, as we were not able to control for the randomness of responding, we refer to our sample as not random and limit statistical inference.

A comparison of the sample and county-level demographics by gender, age group, racial composition and education level revealed dissimilarities. The sample was relatively overrepresented by females, older, white and well-educated participants.

## Measures

### Exposure—Financial Fragility

To measure financial fragility a single question was used (Gupta et al. 2018; Lusardi et al. 2011): ‘Suppose you had an emergency expense that amounts to $400. Based on your current financial situation, how would you pay for this expense?’ (1) temporarily borrow money from friends and family; (2) use the money that is currently in my checking/savings account or with cash; (3) use money from a bank loan or line of credit; (4) use a payday loan, deposit advance or overdraft; (5) sell or pawn something; (6) I would not be able to pay this expense right now; (7) other. Response (2)—indicating having savings to use in case of financial emergency of $400—was indicative of satisfactory financial capability. All other responses were indicative of financial fragility. Consequently, a dichotomous variable was used in the analyses.

It is worth noting that there is no common agreement regarding what value of unexpected expenditures and what time frame is appropriate for the query. The $400 figure has been used in the U.S. following the approach of the Federal Reserve Board (*Report on the Economic Well-Being of U.S. Households in 2017*, 2018) and refers to a very short, yet undefined period of time to come up with cash to cover the expense. Some researchers propose a figure of $2000 for the U.S. economy and €1500 for France, Germany, Italy, Portugal, and the Netherlands (Lusardi et al. 2011), however the longer timeframe (usually 30 days) is suggested when using these larger amounts (Gupta et al. 2018).

### Exposure and Moderator—Financial Control

Financial control was measured with a single question: ‘I feel in control of my finances’, which was a modified version of Furnham’s (1986) economic locus of control variable. Responses 1 = *Not at all*, 2 = *Very little*, and 3 = *Somewhat* were indicative of lack of control, while responses 4 = *Very much* and 5 = *Completely* were indicative of reporting financial control. Consequently, the financial control variable entered the analysis as a dichotomous one.

Outcome Variables.

Despite lack of a single definition of well-being, there is a general agreement that well-being includes emotional (positive: happiness, life satisfaction as well as negative: loneliness, depression), physical (feeling healthy, living longer), and social (social support, friendships) components. Inspired by an outcome-wide approach in which multiple outcomes are considered for a single exposure (VanderWeele 2017a; b), in our study 15 singular well-being outcomes belonging to three distinct conceptual groups, as well as two summary measures were considered. We included in the analysis nine emotional well-being outcomes, four physical well-being outcomes, two social well-being outcomes, as well as an unhealthy days summary measure (Moriarty et al. 2003) and the flourishing index (alpha = 0.905) (VanderWeele et al. 2019; VanderWeele 2017b; Weziak-Bialowolska et al. 2019b) (Table 2).

**Table 2:** Outcomes: questions and response scales

Outcome group	Outcomes	Range
*Singular outcomes*		
Emotional well-being: questions from the flourishing index (VanderWeele 2017a, b; Weziak-Bialowolska et al. 2019b) and the set of healthy days questions of the Health-Related Quality of Life instrument (Moriarty et al. 2003)	Overall, how satisfied are you with life as a whole these days?	0–10—the higher, the better
	In general, how happy or unhappy do you usually feel?	
	Overall, to what extent do you feel the things you do in your life are worthwhile?	
	I understand my purpose in life	
	How would you rate your overall mental health?	
	Thinking about your mental health, which includes stress, depression, and problems with emotions, for how many days during the past 30 days was your mental health not good?	0–30 days
	During the past 30 days, for about how many days:	
	Did you feel sad or depressed?	
	Did you feel worried, tense or anxious?	
	Did you feel very healthy and full of energy?	
Physical well-being: health related questions from the flourishing index (VanderWeele 2017b; Weziak-Bialowolska et al. 2019b) and the set of healthy days questions of the Health-Related Quality of Life instrument (Moriarty et al. 2003)	In general, how would you rate your physical health?	0–10—the higher, the better
	Thinking about your physical health, which includes physical illness and injury, for how many days during the past 30 days was your physical health not good?	0–30 days
	During the past 30 days, for about how many days did pain make it hard for you to do your usual activities, such as self-care, work, or recreation?	
	During the past 30 days, for about how many days have you felt you did not get enough rest or sleep?	
Social well-being: questions from the flourishing index (Campaign to End Loneliness 2016; VanderWeele 2017b; Weziak-Bialowolska et al. 2019b)	I am content with my friendships and relationships	0–10—the higher, the better
	My relationships are as satisfying as I would want them to be	
*Summary measures*		
Unhealthy days summary measure (Moriarty et al. 2003)	Overall number of recent days when physical or mental health was not good (sum of recent days with poor mental health and poor physical health)	0–30 days
Flourishing index (VanderWeele 2017b; Weziak-Bialowolska et al. 2019b)	The flourishing index score is obtained by summing the scores from each of the ten flourishing questions	0–100—the higher, the better

### Control variables

A rich set of control variables was considered when investigating the influence of financial fragility and capability on well-being in our sample. Specifically, we controlled for sociodemographic variables (self-reported; from Wave 1), such as gender, age (below 35, 35–44, 45–54, 55–64, 65 and older), race (White, African American, Other), education (less than high school, high school diploma or equivalent, some college but no degree, associate degree, bachelor’s degree or graduate school education), marital status (married vs. not married), having children at home (yes vs. no) and taking care of an elder (yes vs. no), as it has been empirically confirmed that the above are social determinants of financial conditions (Burgard et al. 2012; Drentea and Lavrakas 2000) and influence changes in emotional, physical, and social well-being (Grossi et al. 2012; Löwe et al. 2008).

Community engagement, volunteering, voting in the last elections and religious service attendance (self-reported; from Wave 1) were also considered in this study because of their associations with well-being (Bloemraad and Terriquez 2016; Lim and Putnam 2010; VanderWeele et al. 2016a, b), and the protective role against financial distress (Krause 1987; Peirce et al. 1996; Renneboog and Spaenjers 2012). Since there is some empirical evidence supporting their discriminatory role, the analysis accounted for labor market status (employed, looking for a job, not working), and perception of financial situation and material deprivation (worrying about expenses, food, safety, and housing) (Arber et al. 2014; Green and Leeves 2013), as well as lifestyle factors (practicing a sport, drinking and smoking [Pawlikowski et al. 2019]). Finally, in each regression, we controlled for the baseline values of the outcome variables (in 2017; Wave 1) to reduce confounding and the possibility of reverse causation. Controlling for the baseline outcome was also employed to correct for individual tendencies to report either high or low scores on outcome variables. The list of control variables with baseline descriptive statistics is presented in Table 1.

#### Statistical analysis

The longitudinal dataset was used to investigate how financial fragility and financial control influence well-being outcomes. An outcome-wide approach was applied, in which multiple outcomes were considered for a single exposure (VanderWeele 2017a). This approach has been theoretically argued (VanderWeele 2017a; VanderWeele et al. 2020b, a) and empirically confirmed as effective in presenting temporal associations across the whole spectrum of outcomes (Białowolski et al. 2019; Chen et al. 2019; Chen and Vanderweele 2018; Kim et al. 2020; Pawlikowski et al. 2019; Steptoe and Fancourt 2020; Węziak-Białowolska et al. 2018). It has the advantage of avoiding cherry-picking of significant results only, limits the risk of p-hacking (Head et al. 2015; Lakens 2015) and seems to facilitate reporting of the so-called ‘negative’ or ‘non-significant’ findings, which has been already proven problematic in scientific publishing due to resistance of journal editors to publish negative results (Fanelli 2010, 2012; Ioannidis 2005; Matosin et al. 2014). Hence, the impact of one’s financial situation (financial fragility and financial control) on well-being outcomes was assessed in 17 separate regressions.

Because of the longitudinal panel data employed, in contrast to many cross-sectional analyses, our approach attempted to offer more plausible evidence for causality by ensuring a logical temporal sequence of cause and effect from the data (VanderWeele et al. 2020a, b; VanderWeele et al. 2016a, b). Specifically, since two exposure variables were measured temporarily prior to outcomes, the intended causal links were more plausible. The temporal association between financial fragility and financial control and well-being outcomes was modeled using three lagged linear regression models:

M1 (financial fragility):

$${WB}_{i,j,2018}={\beta }_{0}+{{{\gamma }_{1}{FF}_{i,2017}+\beta }_{1}X}_{i,2017}{+{\beta }_{2}WB}_{i,j,2017}+{\eta }_{i},\quad i=1,\dots ,N.$$

M2 (perception of financial control):

$${WB}_{i,j,2018}={\beta }_{0}+{{{\gamma }_{2}{FC}_{i,2017}+\beta }_{1}X}_{i,2017}{+{\beta }_{2}WB}_{i,j,2017}+{\eta }_{i} ,\quad i=1,\dots ,N.$$

M3 (moderation effect):

$${WB}_{i,j,2018}={\beta }_{0}+{{{\gamma }_{1}{FF}_{i,2017}+{\gamma }_{2}{FC}_{i,2017}+{\gamma }_{3}{FF}_{i,2017}\bullet {FC}_{i,2017}+\beta }_{1}X}_{i,2017}{+{\beta }_{2}WB}_{i,j,2017}+{\eta }_{i},\quad i=1,\dots ,N.$$

In all specifications subscript *i* represents an individual, subscript *j* represents one out of 17 outcomes, variables *FF*_i,2017_ and *FC*_i,2017_ indicate exposure related to financial fragility and perception of financial control, respectively. *X*_*i,2017*_ is a vector of baseline control variables and *WB*_*i,j,year*_ is one of 17 well-being outcomes—17 independent models were estimated. *β*_*0*_ represents a constant, *β*_*1*_ shows the impact of individual level characteristics (control variables) on a well-being outcome, *β*_*2*_ represents the strength with which a well-being measure in 2017 is related to well-being in 2018, *γ*_*1*_ shows the effect of financial situation on a well-being outcome, *γ*_*2*_—the effect of financial control and *γ*_*3*_—the effect of moderation (interaction). *η*_*i*_ is a disturbance term.

Continuous outcomes were standardized (mean = 0, SD = 1) so that effect estimates could be compared across outcomes.

Since we used a sample that precludes statistical inference for any population beyond ours, we focused on deriving plausible values for the temporal associations between well-being outcomes and either financial control or financial fragility. To this end, the Bayesian linear regression was applied.

Analyses were conducted using Stata 15.

## Results

Among respondents participating in the study, 20.8% could be classed as financially fragile because they reported an inability to cover an unexpected expenditure of $400 from their own resources. However, only 45.3% of respondents declared that they feel in control of their finances (responses: *very much* or *complete*ly) leaving a substantial group of individuals neither financially fragile nor in control of their finances. Among those who felt in control of their finances, only 7.0% were found to be financially fragile, compared to 32.4% of financially fragile respondents among those who did not feel in control. This difference showed that the group of respondents reporting financial fragility and simultaneous control over their finances is small.

Our results suggest that among the participating members of the credit union financial fragility—despite being hypothesized to be harmful for well-being—has a limited negative impact (Table 3). There was only one well-being outcome (out of 17 examined) for which the effect size was moderate, i.e., exceeding 0.15. It was *number of days feeling sad or depressed*. For three additional outcomes the effect sizes were within range 0.10–0.15. These were: (i) *number of days feeling worried, tense, or anxious*, and two items on social well-being: (i) *My relationships are as satisfying as I would want them to be*, and (ii) *I am content with my friendships and relationships*. Thus, only a limited support was found for an association between financial fragility and subsequent well-being, as stated in our RQ1.

**Table 3:** Standardized regression estimates and Bayesian credible intervals (95%) for temporal associations between financial fragility and financial control and well-being (n = 1448)

Outcome	Financial fragility Model M1	Financial control Model M2
*Singular outcomes*		
*Emotional health*		
Life satisfaction	− 0.036 (− 0.096; 0.029)	0.157 (0.070; 0.235)
Happiness	− 0.097 (− 0.167; 0.036)	0.147 (0.055; 0.250)
Meaning in life: Things done in life are worthwhile	− 0.078 (− 0.146; − 0.006)	0.146 (0.094; 0.199)
Purpose in life	− 0.067 (− 0.140; 0.015)	0.092 (0.040; 0.143)
Mental health self-assessment	0.051 (0.005; 0.096)	− 0.007 (− 0.044; 0.031)
Number of days with poor mental health	0.090 (0.014; 0.171)	− 0.157 (− 0.228; − 0.094)
Number of days feeling sad or depressed	0.183 (0.133; 0.235)	− 0.122 (− 0.164; − 0.082)
Number of days feeling worried, tense or anxious	0.110 (0.017; 0.203)	− 0.035 (− 0.104; 0.030)
Number of days feeling very healthy and full of energy	− 0.046 (− 0.157; 0.066)	0.210 (0.145; 0.270)
*Physical health*		
Physical health self-assessment	− 0.035 (− 0.102; 0.037)	0.087 (0.032; 0.145)
Number of days with poor physical health	0.074 (− 0.021; 0.164)	− 0.179 (− 0.228; − 0.131)
Number of days feeling pain that makes it hard to do usual activities, such as self-care, work, or recreation	0.059 (− 0.045; 0.151)	− 0.112 (− 0.211; − 0.020)
Number of days with insufficient rest or sleep	0.070 (− 0.036; 0.167)	− 0.108 (− 0.162; − 0.050)
*Social well-being*		
I am content with my friendships and relationships	− 0.131 (− 0.221; 0.045)	0.090 (0.030; 0.151)
My relationships are as satisfying as I would want them to be	− 0.102 (− 0.171; − 0.033)	0.052 (− 0.020; 0.131)
*Summary measures*		
Unhealthy days summary measure	0.082 (− 0.003; 0.174)	− 0.195 (− 0.270; − 0.106)
Flourishing index	− 0.086 (− 0.152; − 0.015)	0.087 (0.028; 0.144)

The analysis samples were restricted to those with valid data on financial fragility, financial control, well-being outcomes and all other covariates. All models in were adjusted for baseline demographics (gender, age, race, marital status, education level, having children, taking care of an elder), baseline financial situation (labor market status, worrying about expenses, housing, food and safety), baseline civic engagement (volunteering, community engagement, voting), baseline lifestyle factors (religious service attendance, practicing a sport, drinking and smoking). Additionally, each model is controlled for a baseline outcome investigated in a particular model

Regarding financial control, we generally observed that its influence on well-being was larger than the one observed for financial fragility. Having compared the effect sizes for both of these exposures (i.e., financial fragility and financial control) in the case of 12 out of 17 indicators, the effect of financial control for well-being was larger than the effect of financial fragility. Specifically, we found that feeling in control over one’s finances was very favorably associated with the number of days during which one feels very healthy and full of energy, life satisfaction, happiness and meaning in life (effect sizes: β = 0.21, β = 0.16, β = 0.15, β = 0.15, respectively). Financial control was also found to limit the subsequent number of days with poor physical health and poor mental health (effect sizes: β = − 0.18 and β = − 0.16, respectively). We also found that financial control was associated with moderately high effect sizes (above 0.1) with the following three well-being measures: (1) days in which one feels sad or depressed (β = − 0.12), (2) days in which pain makes it difficult to perform everyday activities (β = − 0.11), and (3) days with insufficient rest or sleep (β = − 0.11). Additionally, financial control also revealed higher effect sizes in its temporal association with subsequent the unhealthy days summary measure—one of two composite measures used in the analysis. Consequently, we found more evidence on the promoting role of financial control for emotional well-being, physical well-being, and partially for social well-being which is in line with our RQ2.

Tests for moderation effects revealed very small interaction term for the outcomes that turned out to not to be substantial either for the impact of financial fragility or financial control. This indicated lack of support for the RQ3.

## Discussion

In this article we argued that consequences of one’s financial situation impact well-being in emotional, physical, and social domains. Using longitudinal observational data from the SHINE Well-Being Survey completed by 1448 members of a federal credit union, we examined the prevalence of financial fragility and financial control, their influence on emotional, physical, and social well-being, as well as their impact on composite measures of well-being. We also investigated the role that financial control plays in modifying the relationship between financial fragility and well-being, since it has been shown that people who feel in control are less likely to have excessive debt (Tokunaga 1993; Wahlund and Gunnarsson 1996), demonstrate higher savings (Cobb-Clark et al. 2016), and are also more likely to incur financial risks (Kesavayuth et al. 2018).

Despite the sizeable prevalence of financial fragility, we found less evidence substantiating its effect on well-being than in the case of financial control. The effect sizes in regressions examining the associations between financial fragility and well-being were generally lower than in regression of financial control and well-being. Although this was a bit unexpected, it may be, though, related to the fact that U.S. households can rely on relatively good access to credit and payday loans (Crook 2003; Morse 2011) which, in the occurrence of hardship, can help to alleviate unanticipated financial distress.

Financial control instead was found to play a promoting role for a majority of (positive) emotional well-being outcomes and a protective role against all studied emotional ill-being outcomes. Its promoting role for physical well-being was reflected in improved physical health self-assessment, lower number of days with poor physical health, and lower number of days with insufficient sleep.

Our findings strengthen the reasoning of Kobau et al. (2011) and Seligman (2005, 2008) and recently of VanderWeele (2020a, b) that promoting positive health and human flourishing may require different approaches and interventions than those identified as useful and efficient in reducing ill health and ill-being. Additionally, they underscore the role of financial knowledge that is known to be positively correlated with the perception of financial control, and consequently with more prudent money management (Cobb-Clark et al. 2016; Perry and Morris 2005). They also indirectly corroborate findings of Białowolski et al. (2020) that self-assessed financial literacy—also known as financial confidence—may play an important role for healthy financial decisions and better well-being outcomes. Finally, it can be also argued that financial control translates into long lasting effects (which can be identified in a longitudinal study like ours) and financial fragility has only very immediate consequences and thus does not have a long-term detrimental effect on well-being.

An argument could be made that households experiencing deterioration in health are more likely to take up a loan with an intention to cover their medical expenses and thus a vicious circle of higher debt, higher financial fragility, lower financial control, and lower well-being could arise. Our approach is, however, resilient to this kind of reverse causation because we evaluated well-being outcomes controlling for the baseline levels of well-being and using the exposure variable (financial fragility or financial control) from baseline. This implied that the measurement of financial situation was conducted prior to the observed change in well-being that was an outcome in analysis. Moreover, the issue of debt uptake for medical expenses concerns only a small group of American households—approximately 3% every year (own calculations based on the data from the Panel Study of Income Dynamics 2019). Thus, credit for medical expenses is much less prevalent than financial fragility or insufficient financial control in our sample (20.8% and 54.7%, respectively), which is a further argument that medical debt can be only a minor source of financial fragility or the lack of financial control.

This study is subject to some limitations. First, it made use of observational and nonrandom data and thus the issues of self-selection cannot be completely ruled out and statistical inference cannot be made. Second, although this study controlled for a number of financial behaviors and well-being related factors as potential confounders, there may still exist residual confounding generated by factors, such as traditional personality traits like optimism or neuroticism, for which information was not available. Third, financial control and financial fragility are highly correlated which may reduce the potential to study the interaction between them. Larger samples may be needed to capture individuals who are financially fragile but at the same time who demonstrate high financial control. Finally, the SHINE Health and Well-Being Survey was conducted in a single U.S. county and did not include a nationally representative sample. Therefore, results of this study may not be generalizable to the overall U.S. population or other populations.

Despite its limitations, our study presents relevant policy implications. Usually, the focus of policy is short-term and thus oriented towards mitigating financial fragility. Our results show that not only should financial control be considered as a separate and distinct concept to financial fragility, but also that it could be beneficial to stimulate financial control to increase well-being. Our findings also suggest that direct financial aid provided to people to help them escape the financial fragility trap may not be as effective as equipping them with financial knowledge and increasing their financial control. By enhancing financial control, it may be possible to influence well-being at the later stages of the life course. First, people differ substantially in their approach to wealth accumulation for retirement (Binswanger and Carman 2012). Second, their financial decisions are often myopic. If they result from imprudent behaviors, they have considerable impacts on retirement savings and the quality of life when old (Malone et al. 2010). Third, high levels of self-control stimulate regular savings (Cobb-Clark et al. 2016) and prepare individuals for retirement.

Finally, it is well-known that social and economic inequalities have detrimental effects on people’s lives (Marmot and Allen 2014). In finding solutions, we must learn more about financial behaviors rather than simply examine reactions to financial distress. Simple programs to reduce debts, despite voluntary participation, have been evidenced to be rather ineffective (Karlan and Zinman 2012). By bringing financial skills and financial capability into the analysis of well-being, we recognize not only the importance of material and financial resources for health and well-being promotion but also the immensely important role of financial control.

## Footnotes

1 The issue of reverse causation arises because persons with decreased levels of well-being are more likely to become financially fragile as they are at higher risk to stop working, saving, and repaying their debts.

2 An emblematic behavior of hyperbolic discounting would be a consumer which prefers receiving $100 today over $150 dollars tomorrow but at the same time would choose $150 dollars in two years over $100 in 1 year.

3 SHINE – Sustainability and Health Initiative for NetPositive Enterprise at the Harvard T.H. Chan School of Public Health.

4 Although the community ranked in the second quartile of the 190 listed communities on their five elements of well-being: purpose, social, financial, community, and physical, it ranked in the fourth quintile according to the physical well-being, and in the last quintile according to the financial well-being.
